# Evaluation of computer aided detection of tuberculosis on chest radiography among people with diabetes in Karachi Pakistan

**DOI:** 10.1038/s41598-020-63084-7

**Published:** 2020-04-14

**Authors:** Shifa Salman Habib, Sana Rafiq, Syed Mohammad Asad Zaidi, Rashida Abbas Ferrand, Jacob Creswell, Bram Van Ginneken, Wafa Zehra Jamal, Kiran Sohail Azeemi, Saira Khowaja, Aamir Khan

**Affiliations:** 1 Community Health Solutions, 9th Floor, Al-Tijarah Building, Main Shahrah-e-Faisal, Karachi, Pakistan; 2 London School of Hygiene and Tropical Medicine, Keppel St, Bloomsbury, London, WC1E 7HT UK; 3 Stop TB Partnership, Global Health Campus, Chemin du Pommier 40, 1218 Geneva, Switzerland; 4 Radboud University Medical Center, Radboud, Netherlands; 5 Interactive Research & Development, 4th Floor, Woodcraft Building, Plot No. 3 & 3-A, Sector 47, Korangi Creek Road, Karachi, Pakistan

**Keywords:** Diabetes, Tuberculosis

## Abstract

Pakistan ranks fifth among high tuberculosis (TB) burden countries and also has seventh highest burden for diabetes mellitus (DM). DM increases the risk of developing TB and contributes to adverse TB treatment outcomes hence screening and integrated management for both diseases in high burden countries is suggested. Computer-Aided Detection for TB (CAD4TB) can potentially be used as triage tool in low resource settings to pre-screen individuals for Xpert MTB/RIF testing. The aim of this study was to evaluate the diagnostic accuracy and performance of CAD4TB software in people with diabetes (PWD) enrolled in a TB screening program in Karachi, Pakistan. A total of 694 individuals with a diagnosis of DM (of whom 31.1% were newly diagnosed) were screened with CAD4TB and simultaneously provided sputum for Xpert MTB/RIF testing. Of the 74 (10.7%) participants who had bacteriologically positive (MTB+) results on Xpert testing, 54 (73%) had a CAD4TB score >70; and 155 (25%) participants who tested MTB-negative had scores >70. The area under the receiver operator curve was 0.78 (95% CI: 0.77–0.80). Our study findings indicate that CAD4TB offers good diagnostic accuracy as a triage test for TB screening among PWD using Xpert MTB/RIF as the reference standard.

## Introduction

Diabetes mellitus (DM) increases the risk of developing tuberculosis (TB) up to three fold and may contribute to adverse TB treatment outcomes such as delayed sputum conversion, treatment failure, relapse and death^1^. Given the high proportion of undiagnosed DM in many low and middle-income countries, screening for DM among individuals diagnosed with TB has been recommended^2,3^. Despite World Health Organization (WHO) and the International Union against Tuberculosis and Lung Disease (IUATLD) recommendations for screening followed by integrated management of both diseases, the uptake of screening for TB among people with diabetes (PWD) has remained low^4^.

Recently, there has been renewed interest in the use of chest X-rays (CXR) as a screening tool for TB, particularly in active case finding programs. Computer-Aided Detection for TB (CAD4TB) software provides automatic readings of CXR with a higher score suggestive of TB and has the potential of being used as a triage tool in low-resource settings to pre-screen individuals for Xpert MTB/RIF testing^5^. Pragmatic use of screening through CAD4TB can result in significant savings through reduced Xpert testing specially in lower middle-income countries where high costs of Xpert testing has limited its use. It also reduces the need for an onsite radiologist for immediate readings, which is difficult in low resource settings. Evaluations of the software, using Xpert MTB/RIF as the reference standard have been conducted in Zambia, Bangladesh, Pakistan and most recently in Nepal and Cameroon, reporting a diagnostic accuracy in the range 0.71–0.92^5–8^. Studies of radiographic manifestations of pulmonary TB in PWD report an increased frequency of lower lung field lesions, a higher frequency of cavitation, and more advanced disease^9,10^ yet there is almost no data on use of automated reading software for TB among PWD. Glycemic control also influences radiographic manifestations of pulmonary TB in patients with DM. Therefore, CAD4TB may have variable diagnostic accuracy and lower sensitivity among PWD.

The objective of this study was to evaluate the performance of CAD4TB software in PWD enrolled in a TB screening program in Karachi, Pakistan.

## Results

A total of 694 individuals with a diagnosis of DM [of whom 478 (68.9%) were previously known individuals with DM and 216 (31.1%) were newly diagnosed [based on glucometer random blood sugar (RBS) testing] were screened with CAD4TB and concurrently provided sputum for Xpert MTB/RIF testing. The median age of participants was 54 (IQR 17) years and 374 (53.9%) were male. Of the 74 (10.7%) participants who had bacteriologically positive (MTB+) results on Xpert testing, 47 (63.5%) had a CAD4TB score >80; and 121 (19.5%) participants who tested MTB-negative had scores >80 (Table 1).

**Table 1:** Baseline characteristics of individuals with diabetes, screened with CAD4TB at private TB diagnostic and treatment centers in Karachi, Pakistan from July 2016 till April 2017.

CAD4TB Score	All N (%)	<50 N (%)	50–79 N (%)	>80 N (%)	p-Value
Gender					0.125
Male	374 (53.9%)	119 (51.7%)	153 (51.7%)	102 (60.7%)	
Female	320 (46.1%)	111 (48.3%)	143 (48.3%)	66 (39.3%)	
Age					<0.05
<20	8 (1.1%)	5 (2.2%)	1 (0.3%)	2 (1.2%)	
20–39	92 (13.3%)	47 (20.4%)	27 (9.1%)	18 (10.7%)	
40–59	262 (37.8%)	93 (40.4%)	111 (37.5%)	58 (34.5%)	
>=60	332 (47.8%)	85 (37%)	157 (53%)	90 (53.8%)	
Diabetes Status					<0.001
RBS > 200 mg/dl	216 (31.1%)	116 (50.4%)	54 (18.2%)	46 (27.4%)	
Known DM individuals	478 (68.9%)	114 (49.6%)	242 (81.8%)	122 (72.6%)	
Xpert MTB/ RIF Result					<0.001
MTB Not Detected	620 (89.34%)	223 (96.9%)	276 (93.2%)	121 (72%)	
MTB Detected	74 (10.66%)	7 (3.1%)	20 (6.7%)	47 (28%)	

**Figure 1:**
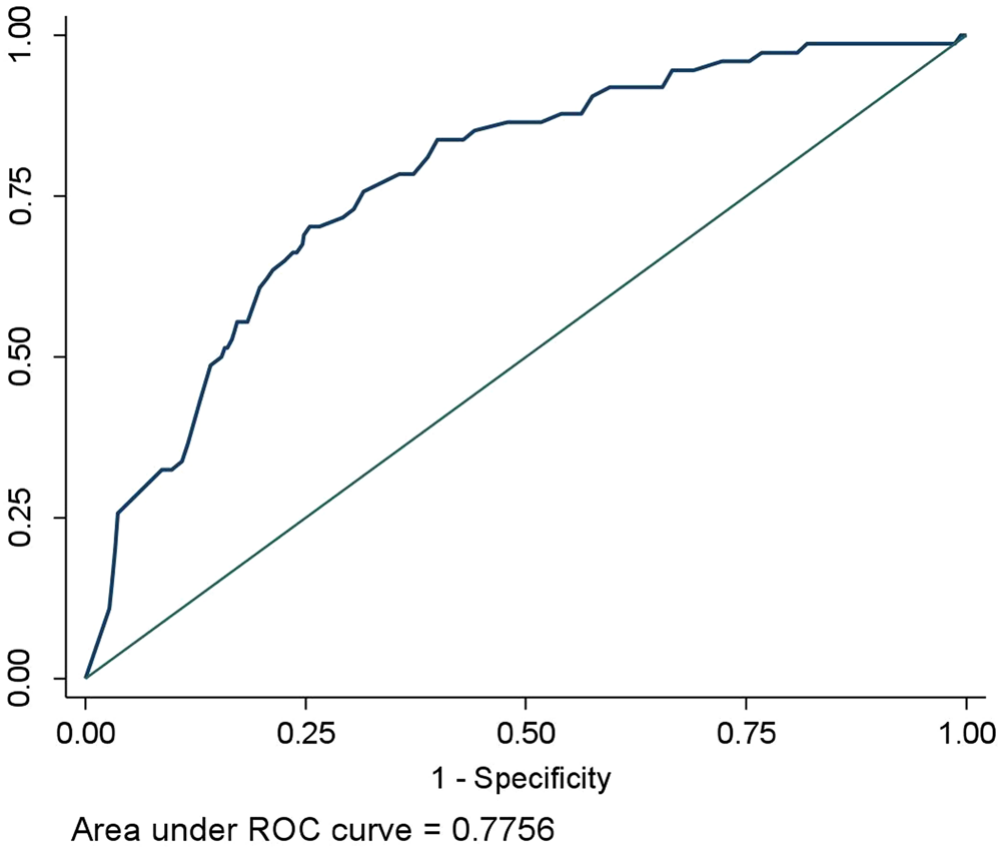
Area under the ROC curve using CAD4TB scores as predictor for MTB detection on Xpert MTB/RIF.

**Figure 2:**
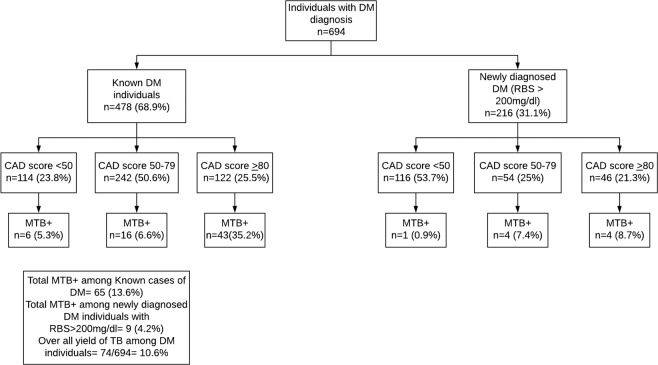
TB testing algorithm among DM diagnosed individuals visiting private TB diagnostic and treatment centers in Karachi, Pakistan (Q3 2016-Q2 2017).

Table 2 shows the sensitivity, specificity, positive predictive value and negative predictive value of CAD4TB score thresholds between 50 and 90, in PWD against the reference standard of Xpert MTB/RIF. Increasing CAD4TB score thresholds improved yield of TB case detection, with a corresponding increase in specificity and decrease in sensitivity. CAD4TB cut-offs of 50 and 90 yielded sensitivities of 90.5% and 48.7% respectively. The potential TB cases missed and Xpert testing yield were the lowest at the cut-off of 50 and highest at 90. Figure 1 shows the receiver operating characteristic curve for CADTB for the study participants using different CAD4TB thresholds. The AUC was 0.78 (95% CI: 0.77–0.80). Among those who were known individuals with DM, 76% individuals had CAD4TB score >50 relative to 46% of those with newly diagnosed DM (Fig. 2).The yield of MTB+ among newly diagnosed DM individuals was 4.2%. The overall yield of TB among DM individuals was 10.6% (Fig. 2).

**Table 2:** Sensitivity, Specificity, Positive predictive Value, Negative Predictive Value at different CAD4TB score thresholds among PWD, tested using Xpert MTB/RIF, visiting TB diagnostic and treatment centers in Karachi, Pakistan (Q3 2016-Q2 2017).

CAD Score	Sensitivity (%)	Specificity (%)	PPV (%)	NPV (%)	No of Xpert tests saved	No of Total Xpert tests	No of TB Cases Missed	MTB+
No triage test	—	—	—	—	—	694		74
Cut-off 50	90.5%	42.4%	15.8%	97.4%	270	424	7	67
Cut-off 60	83.7%	58.6%	19.4%	96.8%	375	319	12	62
Cut-off 70	73.0%	69.5%	22.2%	95.6%	451	243	20	54
Cut-off 80	63.5%	78.7%	26.3%	94.8%	515	179	27	47
Cut-off 90	48.7%	85.8%	29.0%	93.3%	570	124	38	36

**Figure 3:**
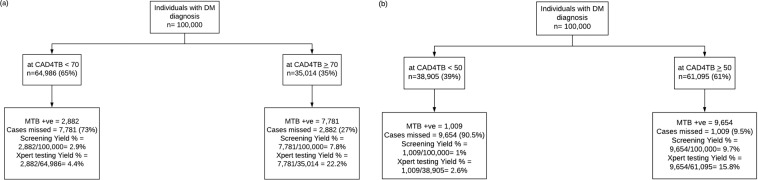
: Comparison of performance of Xpert using a CAD4TB cut off 70 (a) and 50 (b) for a hypothetical population of 100,000 DM diagnosed individuals visiting private TB diagnostic and treatment centers in Karachi, Pakistan.

Figure 3 shows a comparison of the performance of Xpert at CAD4TB score of 70 and 50 for a hypothetical population of 100,000 individuals with DM diagnosis. It indicates that 27% MTB positive cases will be missed when CAD4TB cut off score is >70 in comparison to 9.5% at CAD4TB cut off score >50. The Xpert testing yield at CAD4TB score >70 is higher (22.2%) as compared to CAD4TB cut off score >50 which was 15.8%.

## Discussion

To our knowledge, this is the largest study to date evaluating CAD4TB among PWD and the first study using Xpert MTB/RIF as the reference standard in a programmatic setting. In this screening program, 74 (10.7%) of the 694 PWD undergoing TB testing were diagnosed with bacteriologically confirmed TB. A previous meta-analysis reported a TB prevalence of 0.38–14% among PWD^11^. Our study findings indicate that CAD4TB offers good diagnostic accuracy as a triage test for TB screening among PWD using Xpert MTB/RIF as the reference standard. However, in comparison with the CAD4TB evaluation conducted in Pakistan among individuals with an unknown diabetes status that reported sensitivities in the range 91.0–97.3%, our current study shows lower sensitivities (range 48.6–90.5%) for the corresponding CAD4TB cut-offs on the same version of CAD4TB software on the same version of CAD4TB software^5^. A recent study from Indonesia evaluating CAD4TB among PWD reported an AUC of 0.89 using against the reference standard of bacteriologically and clinically confirmed TB (7 bacteriologically confirmed TB cases and 2 clinically diagnosed)^12^.

With an estimated 19.4 million adults with DM in Pakistan, there is an important opportunity for finding missing cases of TB by integrating TB screening algorithm within routine management of DM patients^13^. This approach, after continuous advocacy by our group is now being included as part of the National Strategic Plan for TB control in Pakistan for implementation at the district-level.

A limitation of the current study was the utilization of patient history and random blood sugar (RBS) to identify PWD instead of more definitive diagnostic tool such as HbA1c which is comparatively more expensive hence unaffordable for most of the visitors in our setting. In addition, the association of glycemic control with CAD4TB scores and MTB positivity was not evaluated in this study. Another limitation of this study is that the symptoms data was not recorded which can be an important factor in risk assessment for CAD scores and enhance the performance of the software^5^.

The utilization of CAD4TB as a triage tool to pre-screen PWD for Xpert MTB/RIF testing can improve case-detection in screening programs and also potentially reduce program costs by promoting more rational use of expensive molecular tests like Xpert MTB/RIF. CAD4TB also enables the use of CXR without the need for expert radiology review with the added benefit of a short turnaround time making it particularly useful in resource-limited settings. Diagnostic algorithms and CAD4TB score cut-offs to be used in PWD need to be carefully appraised along with the cost implications against the number of cases missed in a low resource, high burden population. In addition, the effect of glycemic control on performance of CAD4TB software needs further study.

In conclusion, this study gives a broad overview of the use of CAD4TB tool in settings where universal Xpert MTB/RIF is not feasible to identify patients with DM who are more likely to have a concomitant diagnosis of TB.

## Methods

The study was conducted between July 2016 to April 2017 in 30 private TB treatment and diagnostic centers called “Sehatmand Zindagi” (*Healthy Life*) Centers and in community, mobile X-ray based TB screening camps, located in low middle-income neighborhoods. The static centers and mobile X-ray units are equipped with CAD4TB supported digital CXR and serve as sputum collection points for Xpert MTB/RIF testing. Study participants were part of a broad bi-directional screening program for TB and Diabetes. Individuals reporting to be known cases of DM, currently on DM medication and those who were newly diagnosed with DM, with an RBS > 200 mg/dl on glucometer-based testing were referred for CXR and Xpert MTB/RIF testing.

The CXRs were scored for abnormalities suggestive of pulmonary TB by CAD4TB (version 3.07, Diagnostic Image Analysis Group, The Netherlands). Data analysis was conducted using Stata version 13. Sensitivity and specificity were computed for different CAD4TB score cut-offs using Xpert MTB/RIF as the reference standard and the receptor operator characteristic curves were constructed for CAD4TB.

### Ethics approval and consent to participate

Ethical approval for the study was obtained from the Institutional Review Board of Interactive Research & Development that is registered with the Department of Health and Human Services, USA. Informed consent was taken from the all those undergoing testing under this program and de-identified data was utilized was analysis.

## Data Availability

The datasets used and analyzed during the current study are available from the corresponding author on reasonable request.
